# Time course of the sensitivity and specificity of anti-SARS-CoV-2 IgM and IgG antibodies for symptomatic COVID-19 in Japan

**DOI:** 10.1038/s41598-021-82428-5

**Published:** 2021-02-02

**Authors:** Yuki Nakano, Makoto Kurano, Yoshifumi Morita, Takuya Shimura, Rin Yokoyama, Chungen Qian, Fuzhen Xia, Fan He, Yoshiro Kishi, Jun Okada, Naoyuki Yoshikawa, Yutaka Nagura, Hitoshi Okazaki, Kyoji Moriya, Yasuyuki Seto, Tatsuhiko Kodama, Yutaka Yatomi

**Affiliations:** 1 Department of Clinical Laboratory, The University of Tokyo Hospital, Tokyo, Japan; 2 Department of Clinical Laboratory Medicine, Graduate School of Medicine, The University of Tokyo, 7-3-1 Hongo, Bunkyo-ku, Tokyo, 113-8655 Japan; 3 The Key Laboratory for Biomedical Photonics of MOE At Wuhan National Laboratory for Optoelectronics - Hubei Bioinformatics and Molecular Imaging Key Laboratory, Systems Biology Theme, Department of Biomedical Engineering, College of Life Science and Technology, Huazhong University of Science and Technology, Hubei, People’s Republic of China; 4 Reagent R&D Center, Shenzhen YHLO Biotech Co., Ltd, Shenzhen, Guangdong People’s Republic of China; 5 Business Planning Department, Sales and Marketing Division, Medical and Biological Laboratories Co, Ltd, Tokyo, Japan; 6 Department of Blood Transfusion, The University of Tokyo Hospital, Tokyo, Japan; 7 Department of Infection Control and Prevention, The University of Tokyo, Tokyo, Japan; 8 Department of Gastrointestinal Surgery, The University of Tokyo, Tokyo, Japan; 9 Laboratory for Systems Biology and Medicine, The University of Tokyo, Tokyo, Japan

**Keywords:** Infectious diseases, Diagnostic markers

## Abstract

The accurate and prompt diagnosis of SARS-CoV-2 infection is required for the control and treatment of the coronavirus infection disease 2019 (COVID-19). In this study, we aimed to investigate the time courses of the anti-severe acute corona respiratory syndrome coronavirus 2 (SARS-CoV-2) IgM and IgG titers and to evaluate the sensitivity and specificity of such tests according to the specific day after the onset of COVID-19 among a patient population in Japan. We measured the titers of SARS-CoV-2 IgM and IgG in sera from 105 subjects, including 26 symptomatic COVID-19 patients, using chemiluminescent immunoassay (CLIA) methods utilizing magnetic beads coated with SARS-CoV-2 nucleocapsid protein and spike protein. The results of a ROC analysis suggested the possibility that the cutoff values in Japan might be lower than the manufacturer’s reported cutoff (10 AU/mL): 1  AU/mL for IgM and 5  AU/mL for IgG. The sensitivity of the test before Day 8 after symptom onset was less than 50%; at Days 9–10, however, we obtained a much higher sensitivity of 81.8% for both IgM and IgG. At 15 days or later after symptom onset, the SARS-CoV-2 IgG test had a sensitivity of 100%. These results suggest that if the number of days since disease onset is taken into consideration, these antibody tests could be very useful for the diagnosis of COVID-19 and similar diseases.

## Introduction

Since December 2019, the severe acute respiratory syndrome coronavirus 2 (SARS-CoV-2) has spread throughout the world. The accurate and prompt diagnosis of SARS-CoV-2 infection is required for the control of the COVID-19 pandemic as well as the initiation of adequate individual treatment. In clinical practice, the detection of SARS-CoV-2 RNA using a reverse transcriptase-polymerase chain reaction (RT-PCR) and nasopharyngeal swabs, saliva, or other upper respiratory tract specimens is considered to be the standard method for the diagnosis of COVID-19^1^. Although the specificity of RT-PCR is relatively high, issues persist surrounding insufficient sensitivity (mainly because of the sampling technique), the timing of sample collection, and the types of specimens. Wang et al. reported that sensitivities of RT-PCR for bronchoalveolar lavage specimens, sputum, nasal swabs, fibrobronchoscope brush biopsy, pharyngeal swabs, feces, blood, and urine were 93%, 72%, 63%, 46%, 32%, 29%, 1%, and 0% in a study of 205 COVID-19 patients^2^, and Pasomsub et al. reported that the sensitivity of RT-PCR using saliva samples was 84.2%, compared with RT-PCR using nasopharyngeal and throat swabs^3^. The insufficient sensitivity of the RT-PCR method especially for nasopharyngeal swabs and saliva, which are commonly used specimens, sometimes produces false-negative results. Moreover, the sampling of respiratory and nasopharyngeal specimens can cause secondary infection.

Another method for detecting COVID-19 infection is the measurement of serum titers of virus-specific antibodies to SARS-CoV-2. Compared with the sampling methods required for RT-PCR, this serological assay reduces the risk of aerosol exposure, making it safer for medical staff. Therefore, anti-SARS-CoV-2 IgM and IgG antibodies have been expected to be useful as complementary tests, in addition to RT-PCR, for the diagnosis of COVID-19. However, a limitation of antibody tests is that they require a longer window period after infection than RT-PCR. Actually, the median IgM and IgG seroconversion period has been reported to be 10 days or longer after symptom onset^4–6^. However, information on the early time courses of serum antibody titers and the sensitivity and specificity of antibody tests after the onset of COVID-19 symptoms could make these tests more useful for the COVID-19.

In this study, we aimed to investigate the time courses of the anti-SARS-CoV-2 IgM and IgG titers as measured using chemiluminescent immunoassays (CLIA) and to determine the sensitivity and specificity of these tests according to the specific day after the onset of COVID-19 symptoms in Japanese subjects.

## Results

### Titers of SARS-CoV-2 IgM and IgG

**Figure 1:**
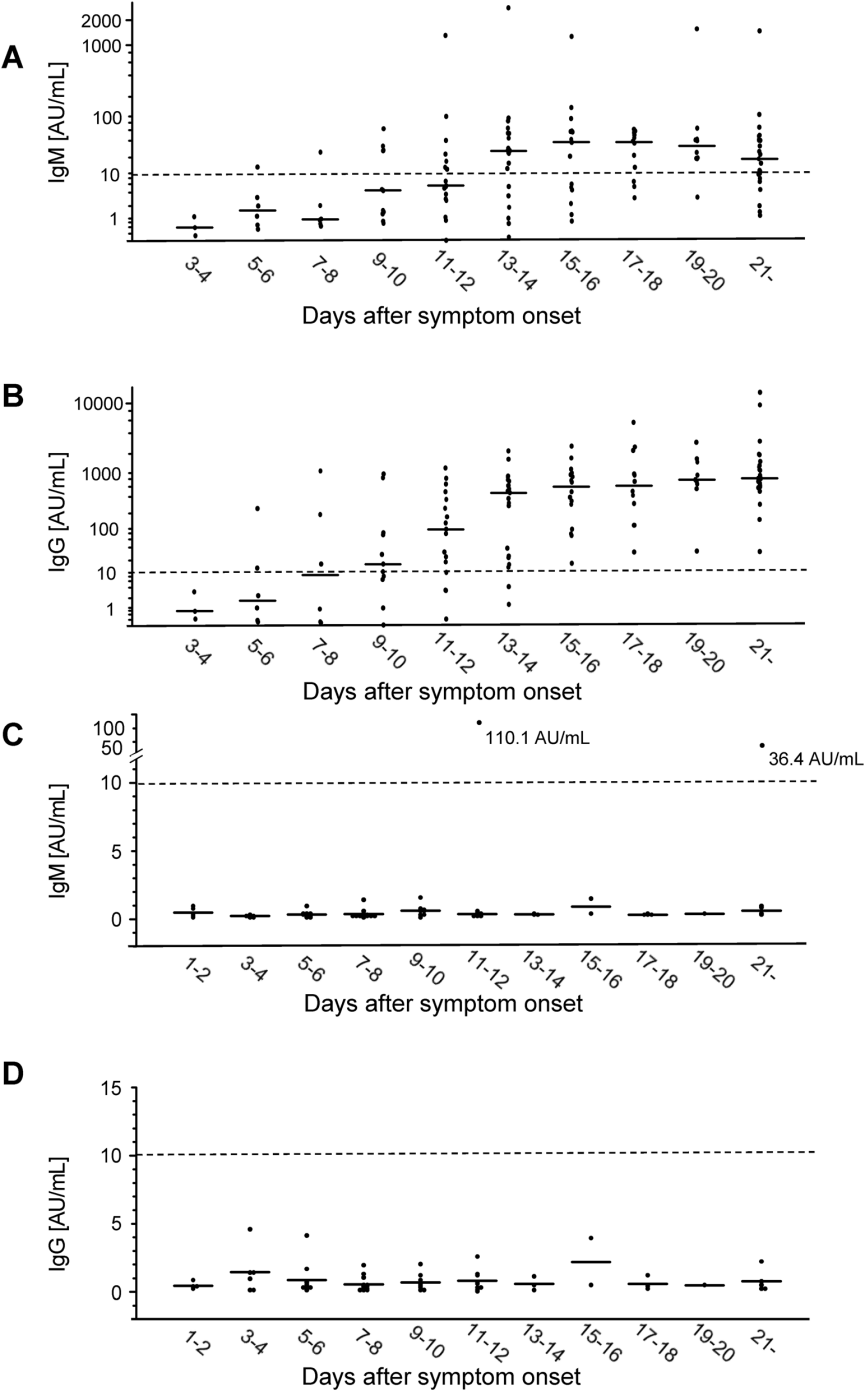
The time courses of serum titers of SARS-CoV-2 antibodies in COVID-19 patients after symptom onset. We measured the titers of SARS-CoV-2 IgM and SARS-CoV-2 IgG in 125 sera collected from RT-PCR-positive patients (n = 26) and 68 sera collected from RT-PCR-negative patients (n = 35). The data are plotted for days 1–2, 3–4, 5–6, 7–8, 9–10, 11–12, 13–14, 15–16, 17–18, 19–20, and ≥ 21 since symptom onset. The bars show the median titers at each timepoint. The broken line shows the manufacturer’s cutoff value (10 AU/mL). (**A**) RT-PCR-positive patients, IgM. (**B**) RT-PCR-positive patients, IgG. (**C**) RT-PCR-negative patients, IgM. (**D**) RT-PCR-negative patients, IgG.

The time courses for the serum titers of SARS-CoV-2 IgM and IgG at each time point (number of days after symptom onset) are shown in Fig. 1. Only data for which information regarding the timing of the sampling (number of days after symptom onset) were available were analyzed. As a result, the titers of SARS-CoV-2 IgM and SARS-CoV-2 IgG were analyzed in the 125 sera collected from RT-PCR-positive patients (n = 26) and in the 68 sera collected from RT-PCR-negative patients (n = 35). To present the intensity of antibody responses after symptom onset, we showed the changes of IgM and IgG titers in each of 26 RT-PCR-positive patients separately in Supplemental Fig. 1. The association between IgM or IgG and COVID-19 severity was shown in Supplemental Fig. 2.

### ROC analysis and Redefinition of the cutoff values

**Figure 2:**
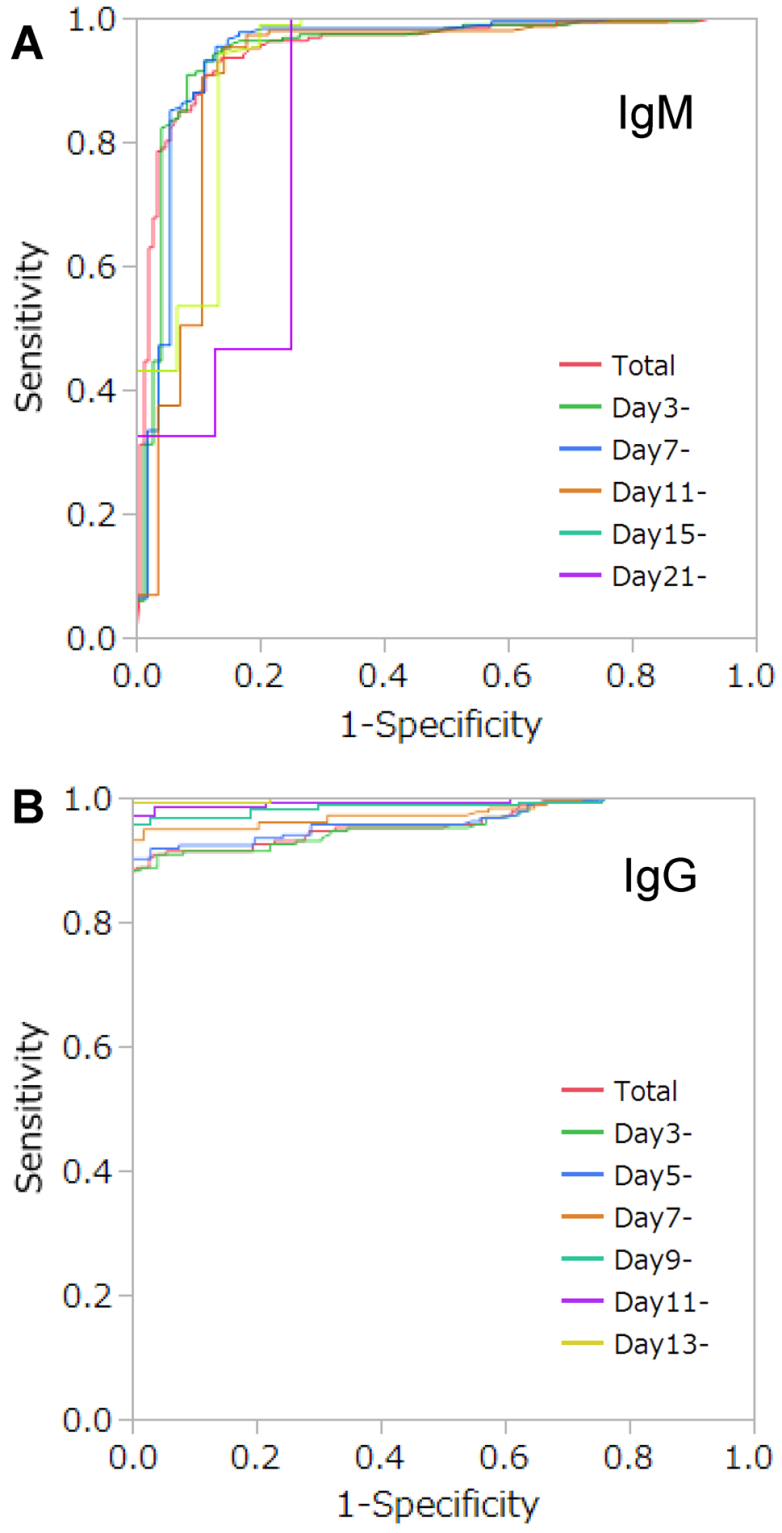
ROC curves for SARS-CoV-2 antibody tests. We compared the discriminating abilities of the SARS-CoV-2 IgM and SARS-CoV-2 IgG titers at different time points after symptom onset. The cutoff value for both tests was set at 10 AU/mL. (**A**) SARS-CoV-2 IgM, (**B**) SARS-CoV-2 IgG.

We performed the ROC analyses at each time point after symptom onset to determine the cutoff values (Fig. 2 and Table 1). When the ROC analyses were performed using all samples we collected (n = 330; RT-PCR-positive n = 186, RT-PCR-negative n = 144), the AUC of IgM and IgG were 0.948 and 0.952, respectively. The redefined cutoff values of both of IgM and IgG were far below the manufacturer’s cutoff value, that’s 0.86 AU/mL and 4.97 AU/mL, respectively. When we calculated the sensitivity and specificity at these cutoff values, the sensitivity and specificity of IgM were 93.0% and 86.8%, respectively. Those of IgG were 88.2% and 100%, respectively.

**Table 1:** The time course of the diagnostic values determined with ROC analyses.

Days after symptom onset	Total	Day3-	Day5-	Day7-	Day9-	Day11-	Day13-	Day15-	Day17-	Day19-	Day21-
**N**											
RT-PCR positive	186	186	183	174	165	147	120	86	67	54	43
RT-PCR negative	144	72	66	54	37	28	18	15	13	9	8
Total	330	258	249	228	202	175	138	101	80	63	51
**IgM**											
AUC	0.948	0.944	0.944	0.942	0.925	0.910	0.931	0.927	0.917	0.875	0.849
[95% CI]	[0.918–0.969]	[0.893–0.972]	[0.888–0.973]	[0.874–0.975]	[0.829–0.969]	[0.786–0.966]	[0.800–0.979]	[0.762–0.980]	[0.717–0.980]	[0.605–0.969]	[0.544–0.964]
Cutoff value [AU/mL]	0.86	0.97	0.97	0.86	1.00	1.00	1.48	1.48	1.19	1.19	1.19
Sensitivity [%]	93.0	90.9	91.8	95.4	94.6	95.2	91.7	94.2	100	100	100
Specificity [%]	86.8	91.7	90.9	87.0	86.5	85.7	88.9	86.7	84.6	77.8	75.0
**IgG**											
AUC	0.952	0.958	0.963	0.975	0.987	0.994	0.998	1.000	1.000	1.000	1.000
[95% CI]	[0.933–0.976]	[0.929–0.975]	[0.936–0.980]	[0.950–0.988]	[0.965–0.995]	[0.974–0.999]	[0.984–0.999]	N.A	N.A	N.A	N.A
Cutoff Value [AU/mL]	4.97	4.97	4.31	4.31	4.31	4.31	4.31	3.94	2.95	2.95	2.95
Sensitivity [%]	88.2	88.2	90.2	93.1	95.8	97.3	99.2	100	100	100	100
Specificity [%]	100	100	100	100	100	100	100	100	100	100	100

Next, we analyzed the data only at specific points after symptom onset. the cutoff values of IgM test varied between 0.86 and 1.48 AU/mL. Regarding IgG test, the AUC reached 1.00 (sensitivity: 100%, specificity: 100%) after Day15, and the cutoff values were equal to the border values between RT-PCR-positive and RT-PCR-negative results (Fig. 2, Table 1). These results suggested the possibility that the cutoff values could be lower than 10 AU/mL in the Japanese population: 1 or 2 AU/mL for IgM and 5 AU/mL for IgG.

To validate these results, we also performed ROC analyses using randomly selected outpatients from April to June 2020 as a control group (n = 105) (Supplemental Fig. 3). The AUCs of the IgM and IgG tests were 0.960 (95% CI 0.931–0.977) and 0.962 (95% CI 0.935–0.978), respectively, and the re-calculated cutoff values for both IgM and IgG were 0.89 AU/mL (sensitivity, 92.5%; specificity, 92.5%) and 3.07 AU/mL (sensitivity, 90.9%; specificity, 97.1%), respectively.

### Changes in diagnostic performance after symptom onset

**Figure 3:**
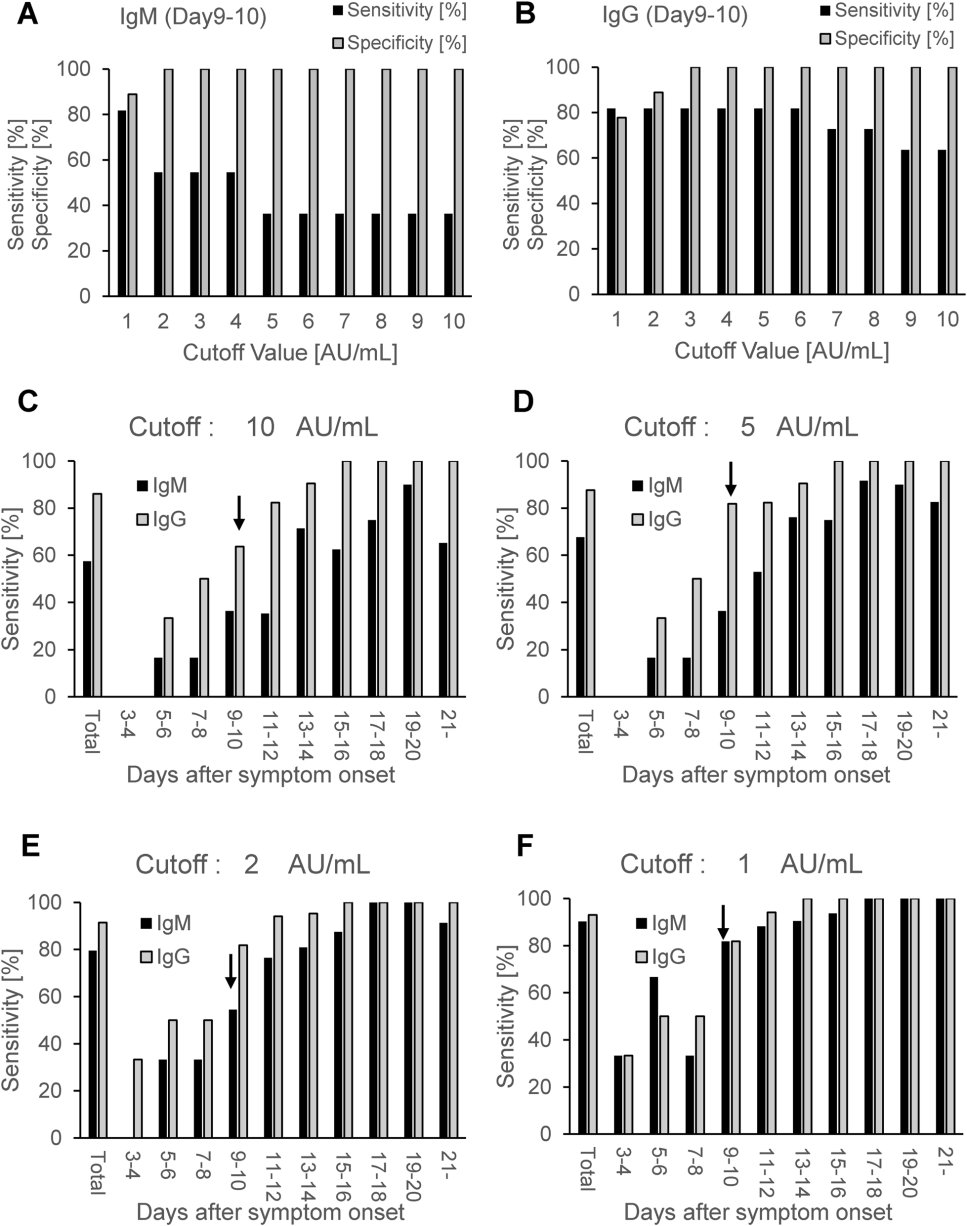
Diagnostic abilities of SARS-CoV-2 antibody tests according to cutoff values and time courses. We compared the sensitivities and specificities for the serum diagnosis of COVID-19 each cut off value (1–10 AU/mL) at 9–10 days after symptom onset (**A**: SARS-CoV-2 IgM, **B**: SARS-CoV-2 IgG) and investigated the time course of the diagnostic ability when the cut off values were set as 1, 2, 5 and 10 AU/mL (**C**: 10 AU/mL, **D**: 5 AU/mL, **E**: 2 AU/mL, **F**: 1 AU/mL).

We calculated the sensitivity and specificity at each cutoff value (1–10 AU/mL). Figure 3A,B shows the changes in sensitivity and specificity when the cutoff values were changed for samples obtained 9–10 days after symptom onset. Especially for IgM, when compared with a cutoff of 5–10 AU/mL, the sensitivity increased up to about 80% at a cutoff of 1 AU/mL. Next, we set the cutoff value for IgM as 1 AU/mL, 2 AU/mL, or 10 AU/mL and that for IgG as 5 AU/mL or 10 AU/mL and compared the sensitivities and specificities among the time points after symptom onset. For an IgM cutoff value of 1 AU/mL (Fig. 3F), the sensitivity at 9–10 days after symptom onset (81.8%) was much higher than that at 7–8 days after symptom onset (33.3%). When the IgM cutoff value was set as 2 AU/mL, the sensitivity at 9–10 days after symptom onset decreased to 54.5% (Fig. 3E). The Youden Index at cutoff values of 1 AU/mL and 2 AU/mL were 0.71 and 0.55, respectively. For an IgG cutoff value of 5 AU/mL, the sensitivity at 9–10 days after symptom onset was 81.8% (Fig. 3D). When the manufacturer’s cutoff value for IgG, 10 AU/mL, was used, the sensitivity at the same time point decreased to 63.6% (Fig. 3C). These results suggest that the serum titers of IgM and IgG after 9 days after symptom onset might have a sufficient sensitivity and specificity if the cutoff values are redefined using the Youden index for the Japanese population.

**Figure 4:**
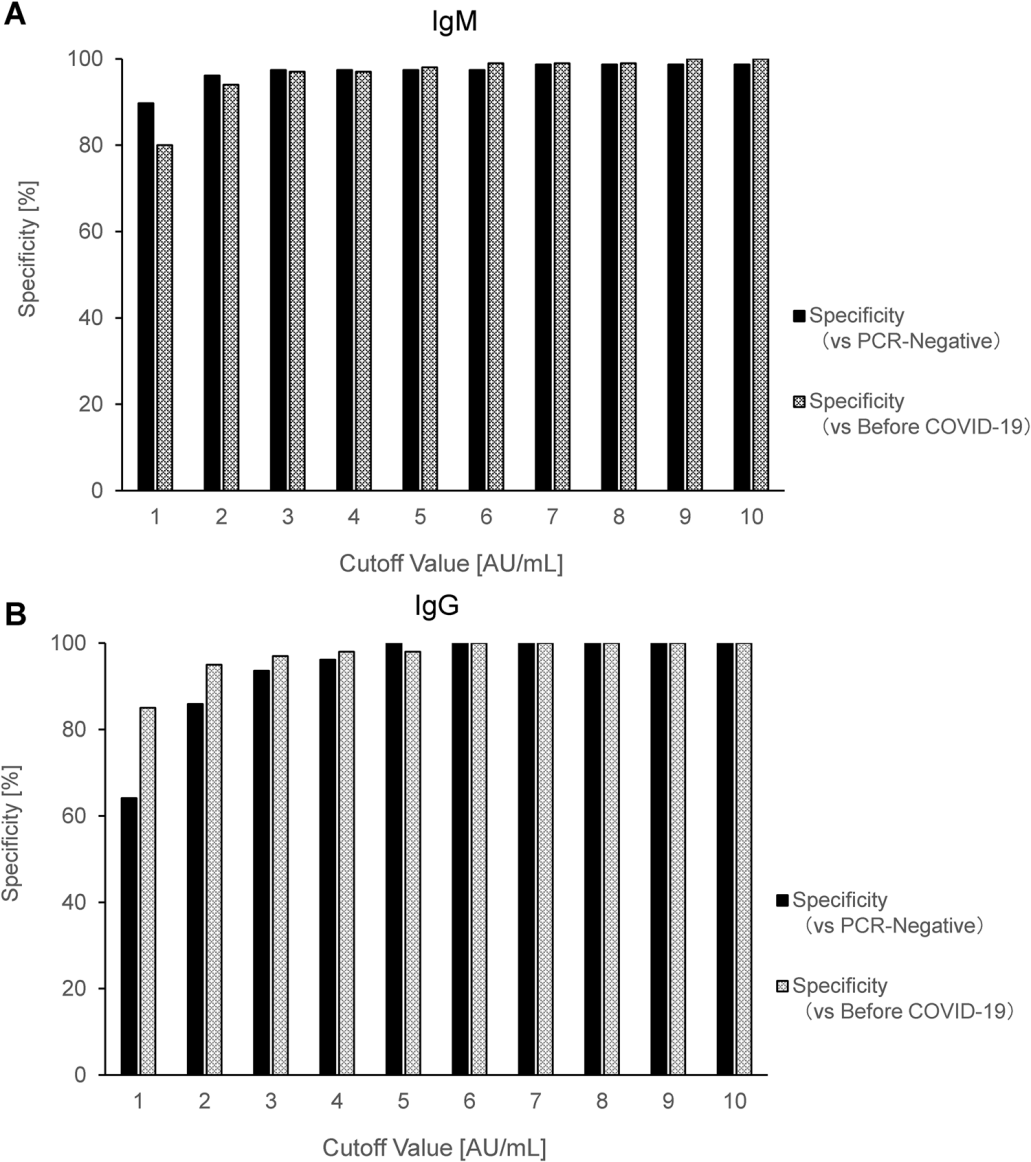
Comparison of the specificity against “RT-PCR-negative group” with that against “Before COVID-19 group”. We compared the specificity against “RT-PCR-negative group” (n = 79) with that against “Before COVID-19 group” (n = 100), which contains healthy subjects in 2017. (**A**) SARS-CoV-2 IgM, (**B**) SARS-CoV-2 IgG.

Although we selected the subjects who had negative results of RT-PCR and had been diagnosed as other diseases, as a negative group, we could not completely exclude the possibility that asymptomatic COVID-19 subjects might be contained in the group. Thus, to validate the results in Fig. 3, we also measured sera collected from healthy subjects in 2017 (n = 100, “Before COVID-19 group”), when SARS-CoV-2 did not exist. We compared the specificity when we used “RT-PCR-negative group” with when we used “Before COVID-19 group” as a negative group (Fig. 4). Regarding IgM, even when the cutoff value was set as the lower value (1 or 2 AU/mL), the specificity against “RT-PCR-negative group” was superior to that against “Before COVID-19 group”. Regarding IgG, when the cutoff value was set from 1 to 4 AU/mL, the specificity against “RT-PCR-negative group” were lower than that against “Before COVID-19 group”. Anyway, we believe that an IgG cutoff value of 5 AU/mL would be still reasonable since the specificities were almost the same when the cutoff value was set at 5 AU/mL or higher, although there remained concerns about the accuracy of titers in the samples collected in 2017, because they were stored at − 20 °C for about 3 years.

## Discussion

In this study, we investigated the time courses of the serum anti-SARS-CoV-2 IgM and IgG titers and evaluated the diagnostic test performance at specific days after the onset of COVID-19. SARS-CoV-2 has four primary proteins: Spike (S), Envelope, Membrane, and Nucleocapsid (N). The higher sensitivity and earlier immune response to the S protein than the N protein has been reported^7^ and antibody against S protein is reportedly more specific than antibody against N protein due to lower cross-reactivity with other coronaviruses^8^. Therefore, the present method which detects both of S and N protein would be more useful for screening method for COVID-19 diagnosis than the antibody tests which detect either of them. In addition, CLIA might be more suitable for clinical testing of SARS-CoV-2 antibody. In a meta-analysis research, which evaluated IgM and IgG tests based on Enzyme-linked immunosorbent assay (ELISA), CLIA, Fluorescence Immunoassays, and Lateral Flow Immunoassays, ELISA- and CLIA-based methods showed higher sensitivity (90–96%) than Fluorescence Immunoassays and Lateral Flow Immunoassays, of which the sensitivity were ranged from 80 to 89%^8^. Considering that ELISA requires a lot of manual operation, whereas CLIA is automated and generally takes less time, CLIA-based assays might be superior from the aspects of sensitivity, safety and throughput.

We confirmed the time courses of the serum antibodies. The median IgM titer increased till Day 18 after symptom onset and then declined (Fig. 1A), while that of IgG remained stable at above 400 AU/mL after Day 13 (Fig. 1B). Similar dynamic changes were also reported by another study^9^. The timing when the median titers of IgM and IgG exceeded the manufacturer’s cutoff value (10 AU/mL) were Day13-14 and Day9-10, respectively, which might be are similar to those for severe acute respiratory syndrome coronavirus 1 (SARS-CoV-1)^10^ and slightly earlier than those for Middle East respiratory syndrome coronavirus (MERS-CoV)^11,12^. In 16 of the 26 RT-PCR-positive patients, the seroconversion of IgG occurred earlier than that of IgM, while the seroconversion of IgM occurred earlier in 2 cases and synchronous seroconversions were observed in 1 case. In 7 cases, we could not determine the seroconversion timing, since both the IgM and IgG titers had already increased above 10 AU/mL even in the earliest available sample (Supplemental Fig. 1). In the usual immunological response to pathogens, IgM is expected to be produced earlier than IgG. Concordant with the present report, Long et al. reported ten subjects in whom the seroconversion of IgG occurred earlier than that of IgM^13^, as has also been reported in other studies conducted in China^14–16^. Although the exact underlying mechanism remains unclear, the earlier seroconversion of IgG might be due to the cross-reactivity of other coronavirus-specific antibodies to the target antigen used in the reagents. Actually, Pinto et al. identified a monoclonal antibody that can neutralize SARS-CoV-2 by engaging the receptor-binding domain of the spike glycoprotein from memory B cells of an individual who had suffered from SARS-CoV-1 infection in 2003^17^. Further studies are necessary to elucidate the mechanisms responsible for the earlier seroconversion of IgG.

We observed one false-positive case in IgM titers (Fig. 1C). This subject was a 2-year-old child with congenital heart diseases, who had symptoms of fever and fatigability, which resembled those of COVID-19. The result of RT-PCR was negative, and the subject was diagnosed as infective endocarditis. IgM titers of the subject were 110.1 AU/mL at Day12 and 36.4 AU/mL at Day24 after the symptom onset, while IgG titers were 1.6 AU/mL and 1.4 AU/mL, respectively. Although the reason for the false positive IgM results remained unknown, repeated blood transfusions might produce some autoantibody that affected the CLIA-based assay.

Regarding the association between antibody titers and the severity of COVID-19, severe group tended to show earlier response in both of IgM and IgG than mild and moderate group (Supplemental Fig. 2). Further study with more subjects is needed to elucidate the association between the immune reaction to SARS-CoV-2 and severity.

Next, we investigated the cutoff values and the time courses of the diagnostic abilities of serum anti-SARS-CoV-2 IgM and IgG titers. When we performed the ROC analyses using the samples at specific days after symptom onset, the AUC of IgM decreased gradually according to the time course, while that of IgG approached 1.0. These changes according to the days after symptom onset were caused by differences in the time courses of the titers; IgG continuously increased, while IgM began to decrease at around 18 days after disease onset. The IgM cutoff values calculated from the present study varied between 0.86 and 1.48 AU/mL over the days since onset, while the cutoff values for IgG were below 5 AU/mL. Consequently, the cutoff values in the Japanese population could be lower (1 or 2 AU/mL for IgM and 5 AU/mL for IgG) than the manufacturer’s cutoff value (10 AU/mL). In previous studies in which the same CLIA-based assays were used, the false-positive rates for the IgM and IgG tests were reported to be 0% (0/33) and 9.1% (3/33) in an Italian study^9^ and 9.1% (4/44) and 0% (0/44) in a Chinese study^18^, respectively, which are somewhat higher than those in the present study (1.3% [1/79] and 0% [0/79]). One of the mechanisms for false-positive results might be the presence of autoimmune diseases, and the prevalences of autoimmune diseases differ among population. For example, the prevalence of SLE, which reportedly causes false-positive cases, in Italy, China and Japan are reportedly 40–81, 10–30^19^ and 3.7–19.1^20^ per 100,000 of the population, respectively. In this study, RT-PCR negative group included subjects who were complicated with autoimmune diseases (Table 2B). The highest titers in these subjects were 1.55 AU/mL for IgM and 4.28 AU/mL for IgG, which were under the redefined cutoff values (2 AU/mL for IgM and 5 AU/mL for IgG).

**Table 2:** Characteristics of the subjects.

**(A) RT-PCR positive**		
Number of subjects	Total	26
	M	21
	F	5
Age [years] (range)		68.0 (24–89)
Severity	Mild	4
	Moderate	13
	Severe	9
**(B) RT-PCR negative**		
Number of subjects	Total	79
	M	56
	F	23
Age [years] (range)		67.5 (2–95)
Diagnosis	Bacterial pneumonia	24
	Fungal pneumonia	3
	Aspiration pneumonia	20
	Interstitial pneumonia	4
	Septic shock	2
	Cardiogenic shock	6
	COPD	2
	Others	18
Complications of autoimmune disease	RA	5
	SLE	2
	APS	1
	Sjögren's syndrome	3
	IgG4-related disease	1
	Scleroderma	2
	Myasthenia gravis	1

*APS* anti-phospholipid antibody syndrome, *COPD* chronic obstructive pulmonary disease, *RA* rheumatoid arthritis, *SLE* systemic lupus erythematosus.

Regarding the time course of sensitivity, the diagnostic ability of antibodies, especially IgG, increases as time passes from the onset of symptoms. Zhao et al. reported that RT-PCR had a higher sensitivity (66.7%) than IgM (28.7%) and IgG (19.1%) within 8 days from the onset of symptoms, while the sensitivity of RT-PCR decreased to 54.0% after Day 8 and that of the antibody tests increased^4^. Concordantly, the diagnostic performances of both IgM and IgG were insufficient at the time points before Day 7–8 (Fig. 3C–F), while at Day9-10 we obtained much higher sensitivity when set on lower cutoff values; For IgM, the sensitivity at the cutoff value of 10 AU/mL, 2 AU/mL and 1 AU/mL were 36.4%, 54.5% and 81.8%, respectively and for IgG, the sensitivity at the cutoff value of 10 AU/mL and 5 AU/mL were 63.6% and 81.8%, respectively. These results suggested that setting the optimal cutoff value for Japanese population could make the serum antibody test more useful tool for diagnosis of COVID-19 infection, although further studies are necessary to prove this hypothesis.

It was of note the IgG titers of all the sera collected from RT-PCR-positive patients (n = 26) were above 10 AU/mL at 15 days or later after symptom onset (Fig. 1B), and the sensitivity was 100%. Several subjects with non-COVID-19 diseases, such as pneumonia caused by other pathogens or collagen diseases, can show similar clinical course and CT results, which sometimes makes a differential diagnosis difficult. The high sensitivity of IgG after Day 15 could help physicians to rule out COVID-19.

A limitation of this study is that only symptomatic patients were examined. Recently, the asymptomatic COVID-19 subjects have been reported to have antibody titers below the cutoff values^21^. According to a recent report, T cell immunity is sufficient to exclude SARS-CoV-2 in asymptomatic patients^22^. Therefore, in respect to infection control, which requires the screening of asymptomatic subjects, an antibody test alone might be insufficient, and combination with a PCR-based test is desirable. Nevertheless, antibody tests could help physicians to diagnose COVID-19 or non-COVID-19 presenting with similar clinical courses among symptomatic subjects.

In summary, we investigated the time courses of the diagnostic performances of SARS-CoV-2 antibody tests in Japan and observed a high sensitivity after Days 9–10. Considering the high sensitivity of IgG levels at 15 days after symptom onset, the IgG test could be a very useful diagnostic tool for ruling out the possibility of COVID-19.

## Materials and methods

### Samples

We collected residual serum samples remaining after routine clinical testing from 105 subjects who underwent RT-PCR testing at The University of Tokyo Hospital. Of these 105 subjects, 26 were diagnosed as having COVID-19 based on the results of RT-PCR, and the remaining 79 subjects were considered to have negative results. The characteristics of the subjects are described in Table 2. We categorized the RT-PCR-positive subjects into three groups: those requiring no oxygen therapy (mild group), those requiring oxygen treatment without mechanical respiratory ventilation support (moderate group), and those requiring mechanical respiratory ventilation support (severe group). The RT-PCR-negative subjects had symptoms such as fever, cough or dyspnea and COVID-19 was denied by the negative results of RT-PCR. They were finally diagnosed as other diseases such as bacterial pneumonia, aspiration pneumonia, septic shock, cardiogenic shock, chronic obstructive pulmonary disease. Serum specimens were stored at − 80 °C and were centrifuged at 2300×*g* for 5 min before measurement.

The current study was performed in accordance with the ethical guidelines of the Declaration of Helsinki. Participants were informed about the study and informed consent was obtained in the form of an opt-out on the website. Patients who rejected to be enrolled in our study were excluded. The study design was approved by The University of Tokyo Medical Research Center Ethics Committee, which waived written informed consent because archived specimens were used and data in this retrospective study were retrieved from medical records (2019300NI-3). Several patients enrolled in the present study have been included in other report^23^.

### Methods

The serum titers of SARS-CoV-2 IgM and IgG were measured using the iFlash3000 fully automatic CLIA analyzer from YHLO Biotechnology Company, Ltd. (Shenzhen, China). We used SARS-CoV-2 IgM and SARS-CoV-2 IgG kits containing magnetic beads coated with SARS-CoV-2 N protein and S protein. The performance of this assay has been validated at 10 hospitals in China^24^. According to the manufacturer’s insert, the cutoff value for the detection of both IgM and IgG is 10 AU/mL.

### Analysis

Receiver operating characteristics (ROC) analyses were performed using JMP PRO v15.0 (SAS Institute Inc., Cary, NC). The area under the curve (AUC) was expressed as the median and 95% confidence interval (CI). For each ROC curve, we redefined the cutoff values so that the Youden Index (= Sensitivity + Specificity – 1) was at a maximum.

## Supplementary Information

Supplementary Information.

## Data Availability

The datasets generated or analyzed in the current study are available upon reasonable request.

## Abbreviations

AUC: Area under the curve
APS: Anti-phospholipid antibody syndrome
CLIA: Chemiluminescent immunoassay
COPD: Chronic obstructive pulmonary disease
COVID-19: Coronavirus infection disease 2019
ELISA: Enzyme-linked immunosorbent assay
MERS-CoV: Middle east respiratory syndrome coronavirus
N protein: Nucleocapsid protein
RA: Rheumatoid arthritis
ROC: Receiver operating characteristics
RT-PCR: Reverse transcriptase-polymerase chain reaction
SARS-CoV-1: Severe acute respiratory syndrome coronavirus 1
SARS-CoV-2: Severe acute respiratory syndrome coronavirus 2
SLE: Systemic lupus erythematosus
S protein: Spike protein
