# Supplementary material for: Time course of the sensitivity and specificity of anti-SARS-CoV-2 IgM and IgG antibodies for symptomatic COVID-19 in Japan
Source: Sci Rep. 2021 Feb 2;11:2776. doi: 10.1038/s41598-021-82428-5 (PMC7854735; doi:10.1038/s41598-021-82428-5)
Supplement: Supplementary file 1 — Supplementary Information. [file 41598_2021_82428_MOESM1_ESM.pdf]

Time course of the sensitivity and specificity of anti-SARS-CoV-2 IgM and IgG antibodies for symptomatic COVID-19 in Japan

Yuki Nakano[1], Makoto Kurano[1,2*], Yoshifumi Morita[1], Takuya Shimura[1], Rin Yokoyama[1], Chungen Qian[3], Fuzhen Xia[4], Fan He[4], Yoshiro Kishi[5], Jun Okada[5] , Naoyuki Yoshikawa[1], Yutaka Nagura[6], Hitoshi Okazaki[6], Kyoji Moriya[7], Yasuyuki Seto[8], Tatsuhiko Kodama[9], and Yutaka Yatomi[1,2*]

[1]Department of Clinical Laboratory, the University of Tokyo Hospital, Tokyo, Japan

[2]Department of Clinical Laboratory Medicine, Graduate School of Medicine, the University of Tokyo, Tokyo, Japan

[3]The Key Laboratory for Biomedical Photonics of MOE at Wuhan National Laboratory for Optoelectronics - Hubei Bioinformatics & Molecular Imaging Key Laboratory, Systems Biology Theme, Department of Biomedical Engineering, College of Life Science and Technology, Huazhong University of Science and Technology, Hubei, P.R. China

[4]Reagent R&D Center, Shenzhen YHLO Biotech Co., Ltd, Guangdong, P.R. China

[5]Business Planning Department, Sales & Marketing Division, Medical & Biological Laboratories Co., Ltd, Tokyo, Japan

[6]Department of Blood Transfusion, the University of Tokyo Hospital, Tokyo, Japan

[7]Department of Infection Control and Prevention, The University of Tokyo, Tokyo, Japan

[8]Department of Gastrointestinal Surgery, The University of Tokyo, Japan

[9]Laboratory for Systems Biology and Medicine, The University of Tokyo, Tokyo, Japan

*Corresponding author

Running Title: COVID-19 diagnosis using serum IgM and IgG

Corresponding author:

Makoto Kurano, MD, PhD and Yutaka Yatomi, MD, PhD

Department of Clinical Laboratory Medicine, Graduate School of Medicine,

The University of Tokyo, 7-3-1 Hongo, Bunkyo-ku, Tokyo 113-8655, Japan

Tel: +81-3-3815-5411; Fax: +81-3-5689-0495;

E-mail: kurano-tky@umin.ac.jp (M.K.), yatoyuta-tky@umin.ac.jp (Y. Y.)

Supplemental Fig. 1

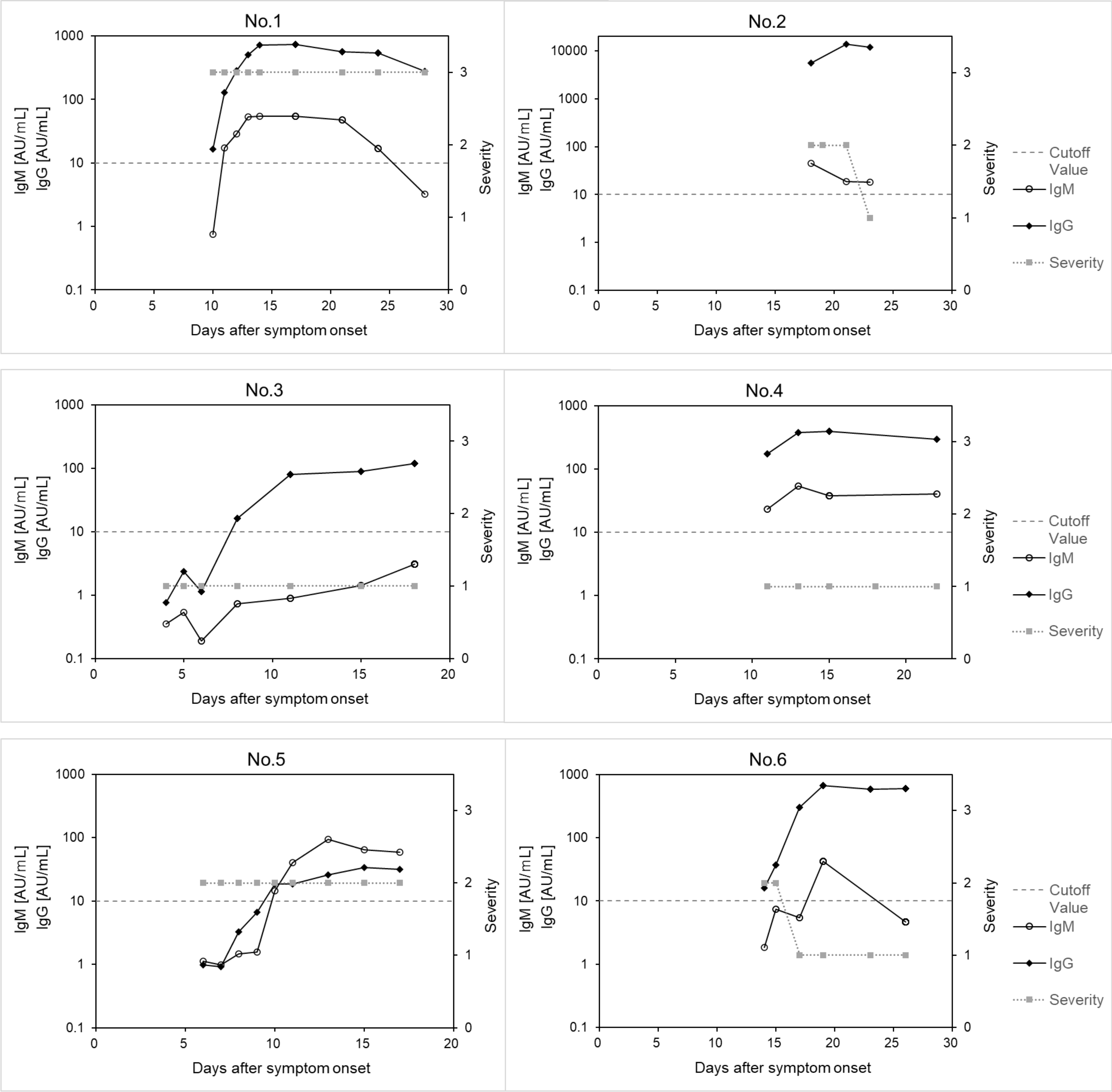

Supplemental Figure 1. Time courses of IgM and IgG in 26 RT-PCR positive cases.

Time courses of the titers of SARS-CoV-2 IgM and SARS-CoV-2 IgG in 26 RT-PCR-positive patients. Gray dashed line shows the severity (1, mild; 2, moderate; 3, severe) on each sampling day.

Supplemental Fig. 1 (continued)

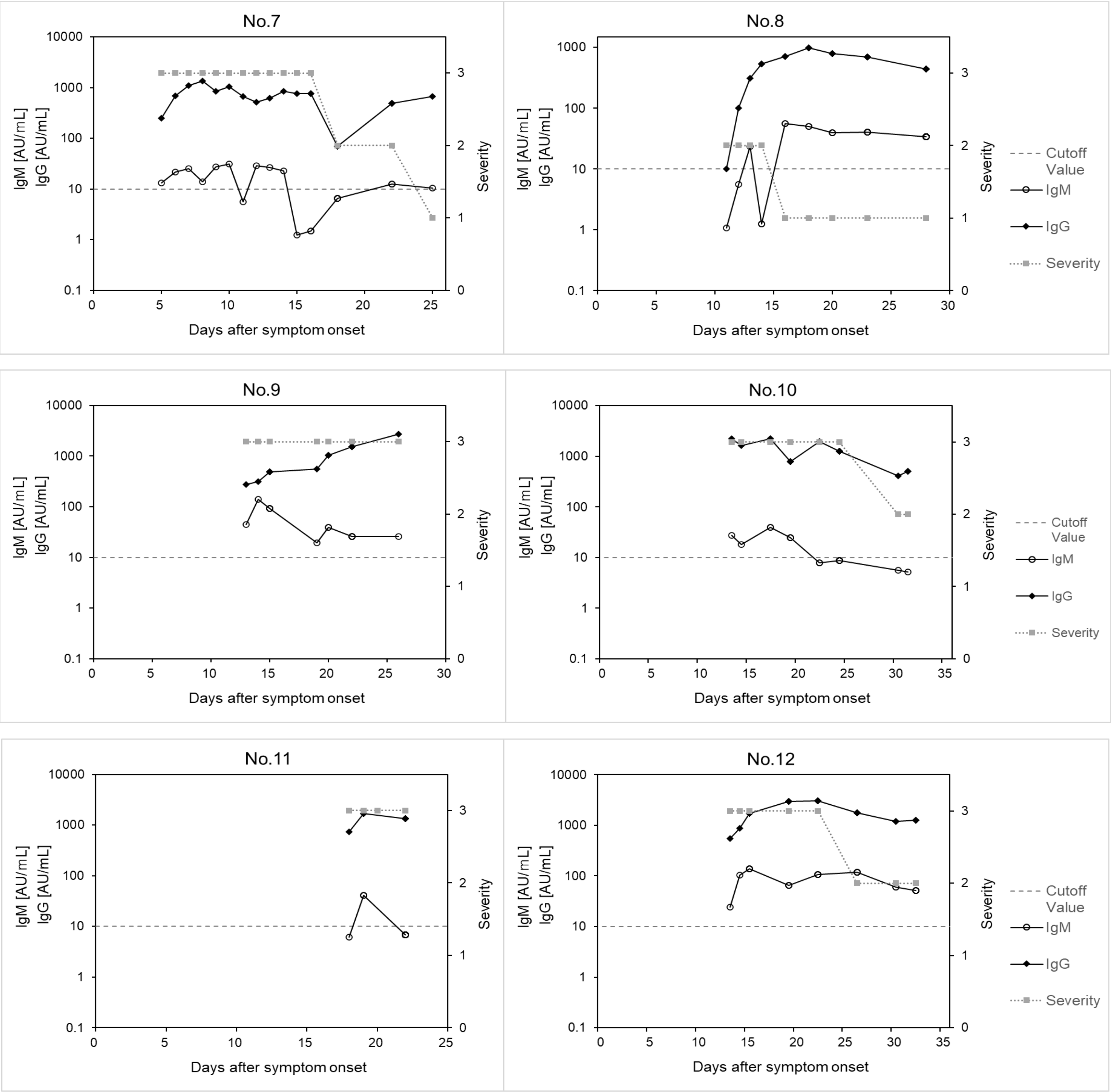

Supplemental Figure 1. Time courses of IgM and IgG in 26 RT-PCR positive cases.

Time courses of the titers of SARS-CoV-2 IgM and SARS-CoV-2 IgG in 26 RT-PCR-positive patients. Gray dashed line shows the severity (1, mild; 2, moderate; 3, severe) on each sampling day.

Supplemental Fig. 1 (continued)

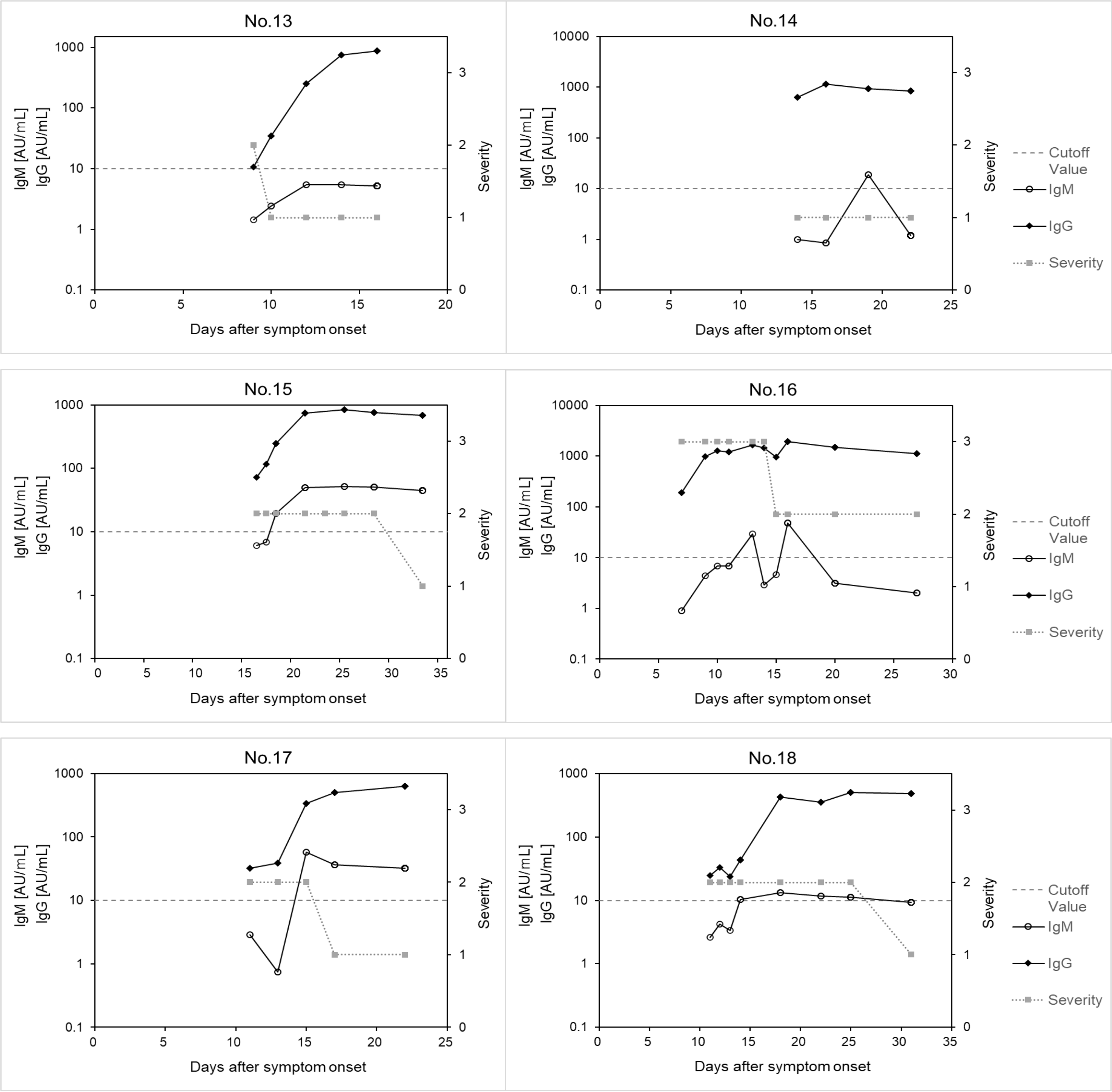

Supplemental Figure 1. Time courses of IgM and IgG in 26 RT-PCR positive cases.

Time courses of the titers of SARS-CoV-2 IgM and SARS-CoV-2 IgG in 26 RT-PCR-positive patients. Gray dashed line shows the severity (1, mild; 2, moderate; 3, severe) on each sampling day.

Supplemental Fig. 1 (continued)

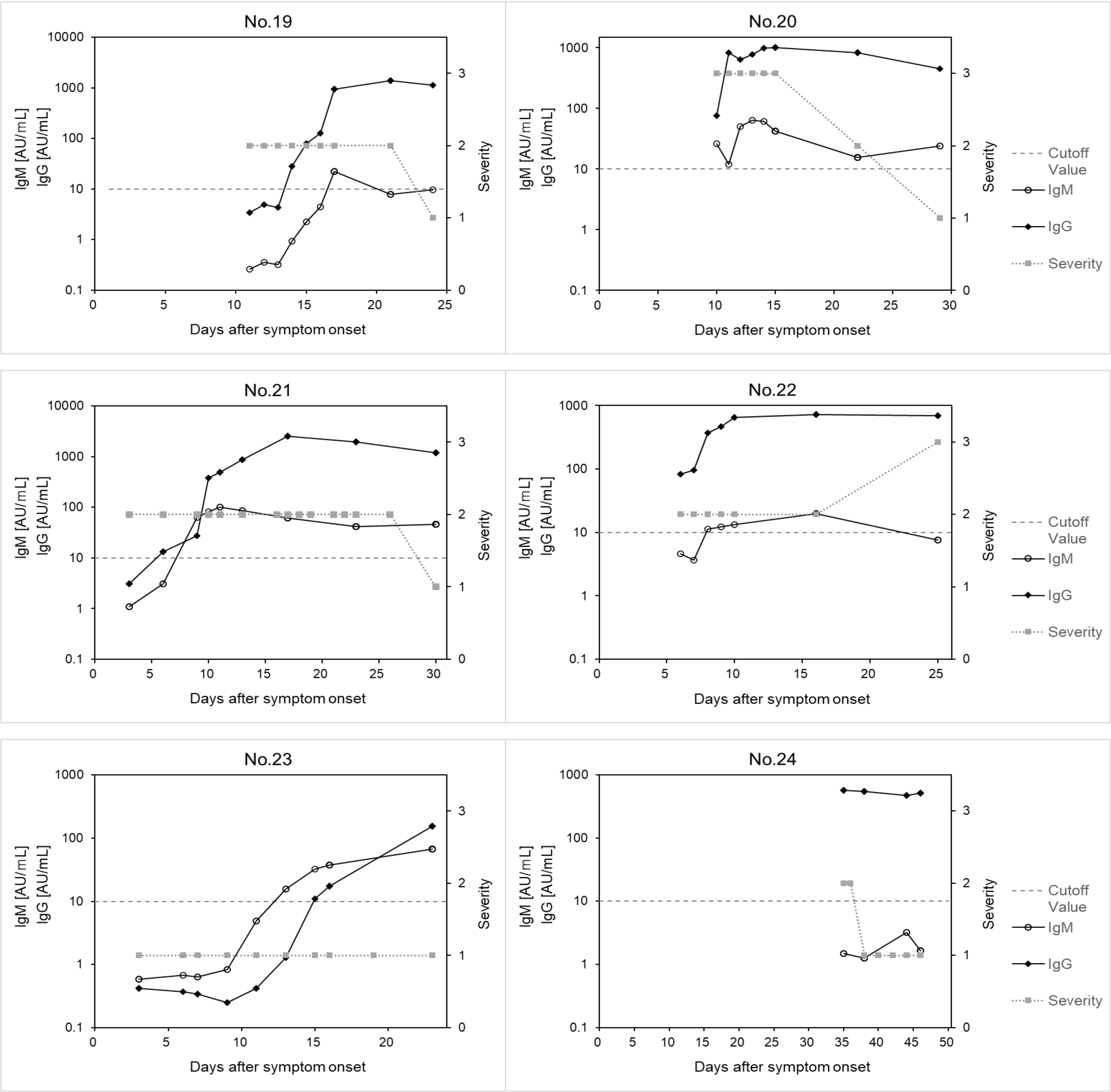

Supplemental Figure 1. Time courses of IgM and IgG in 26 RT-PCR positive cases.

Time courses of the titers of SARS-CoV-2 IgM and SARS-CoV-2 IgG in 26 RT-PCR-positive patients. Gray dashed line shows the severity (1, mild; 2, moderate; 3, severe) on each sampling day.

Supplemental Fig. 1 (continued)

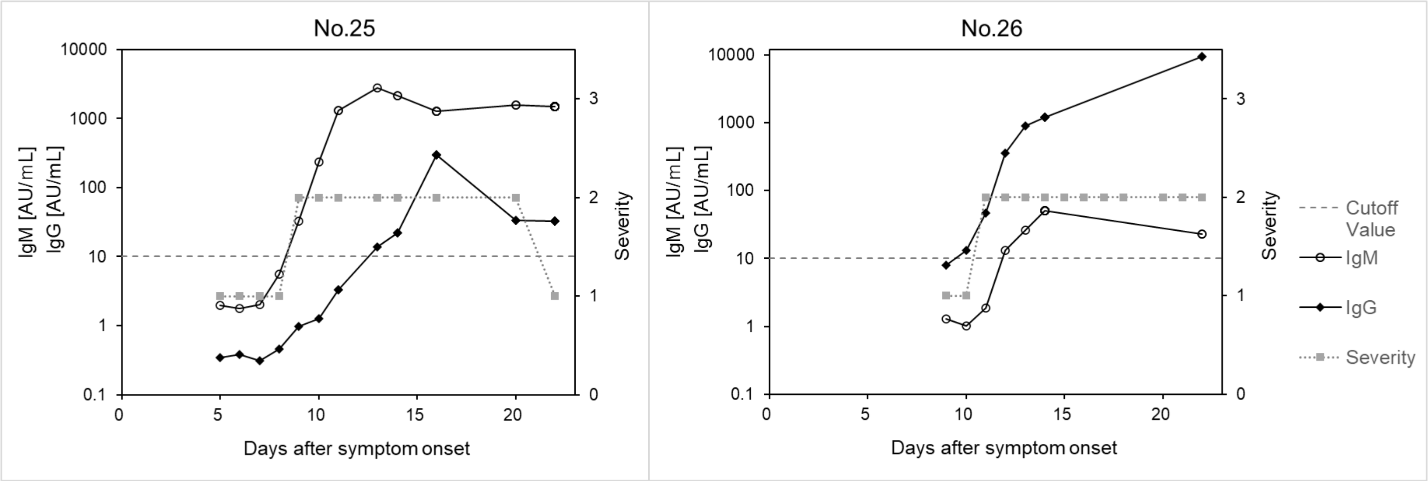

Supplemental Figure 1. Time courses of IgM and IgG in 26 RT-PCR positive cases.

Time courses of the titers of SARS-CoV-2 IgM and SARS-CoV-2 IgG in 26 RT-PCR-positive patients. Gray dashed line shows the severity (1, mild; 2, moderate; 3, severe) on each sampling day.

Supplemental Fig. 2

A

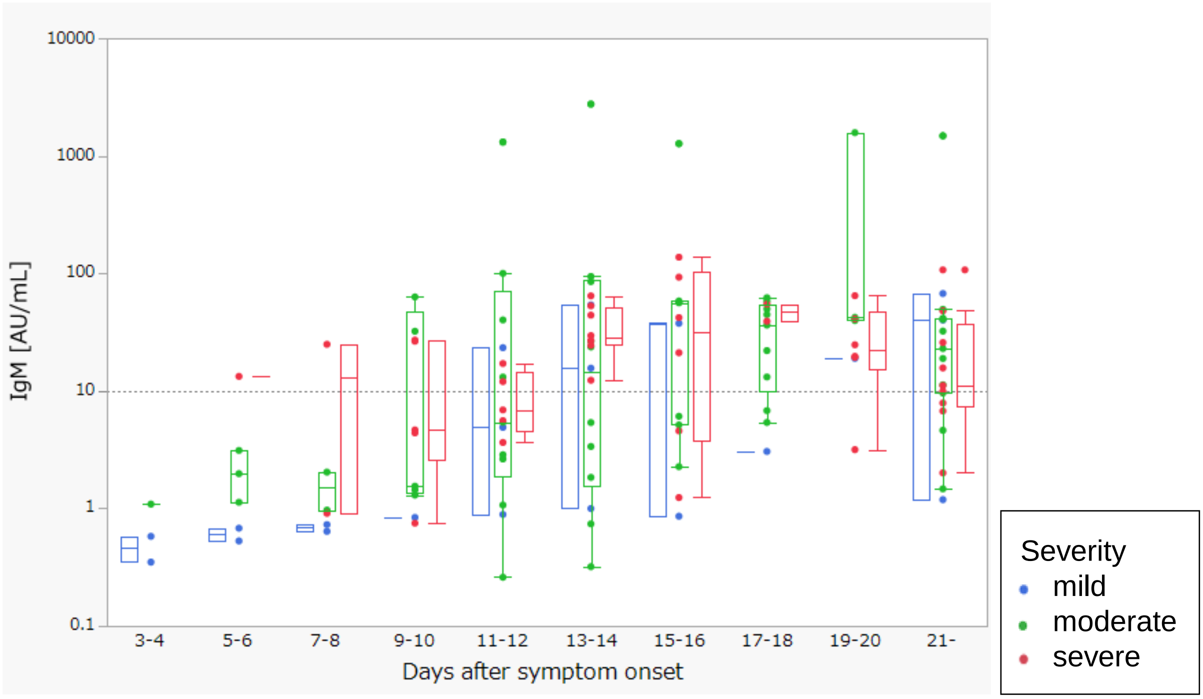

B

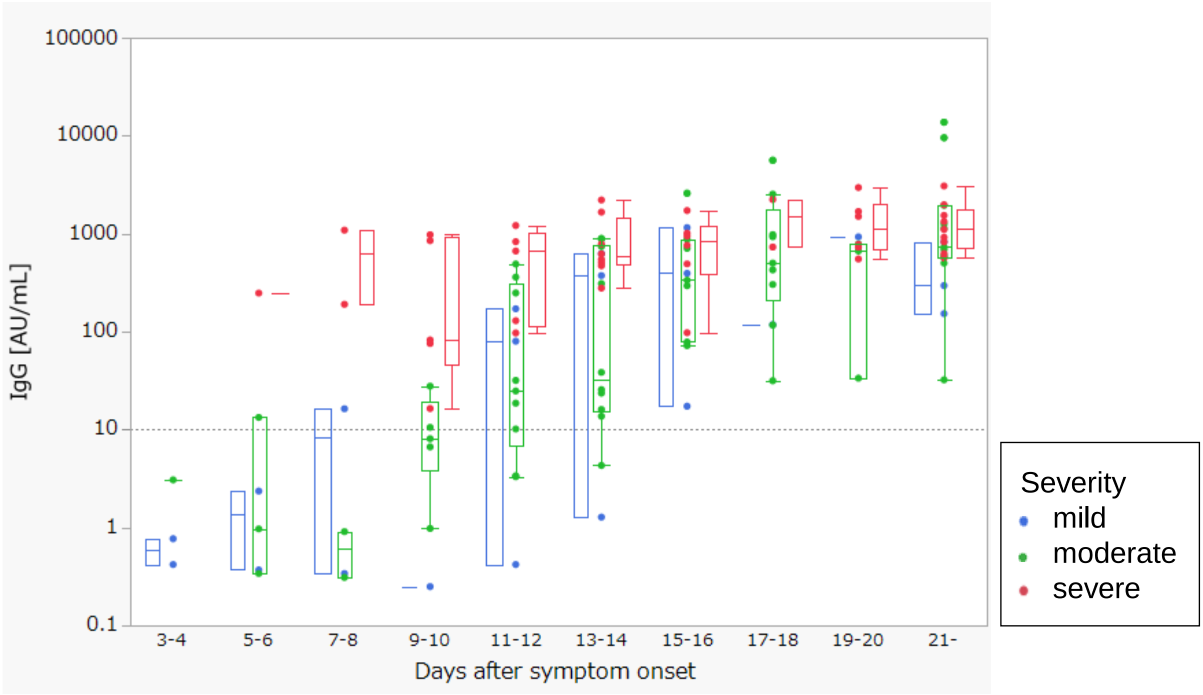

Supplemental Figure 2. Association between the time courses of IgM and IgG titers and the COVID-19 severity.

The association was shown between the titers of SARS-CoV-2 IgM and SARS-CoV-2 IgG in 125 sera collected from RT-PCR-positive patients (n = 26) and the COVID-19 severity (mild, moderate and severe).

Supplemental Fig. 3

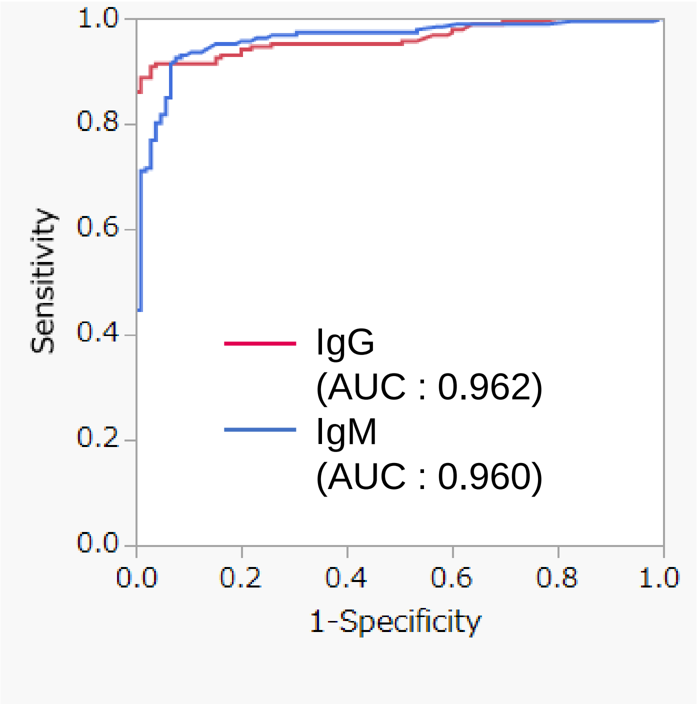

Supplemental Figure 3. ROC curves of the SARS-CoV-2 antibody tests.

We compared the discriminating ability of the titer of SARS-CoV-2 IgM and SARS-CoV-2 IgG using RT-PCR-positive samples (n = 186) and the subjects we randomly selected from outpatients from April to June 2020 as control group (n = 105).
